# Perichondritis: inspect the lobule

**DOI:** 10.1186/s12245-020-00310-z

**Published:** 2020-10-28

**Authors:** Eli Bress, Jason E. Cohn

**Affiliations:** 1 Department of Otolaryngology-Head and Neck Surgery, Philadelphia College of Osteopathic Medicine, 4190 City Line Avenue, Philadelphia, PA 19131 USA; 2 Department of Otolaryngology-Head and Neck Surgery, Division of Facial Plastic Reconstructive Surgery, Louisiana State University Health Sciences Center, 1501 Kings Highway, Shreveport, LA 71103 USA

**Keywords:** Perichondritis, Auricle, Pinna, Ear, Otitis externa

## Abstract

**Case presentation:**

This is a brief report of a 57-year-old Caucasian female presented with a 4-day history of worsening left ear pain. Her symptoms began with left otalgia and otorrhea which progressed to helical erythema, prompting a visit to the emergency department. She was noted to have erythema of the left auricle and swelling of the left auditory meatus. Our otolaryngology service observed erythema of the auricle with sparing of the lobule.

**Diagnosis:**

The diagnosis to be otitis externa with perichondritis was established, and we recommended otic ciprofloxacin-hydrocortisone, IV vancomycin, and ciprofloxacin. The patient had marked improvement and was discharged on an oral and otic fluoroquinolone. In this case, the diagnosis of perichondritis was made by a classic physical examination finding: erythema and edema with sparing of the fatty lobule. This key finding helps to distinguish perichondritis from otitis externa.

## Case presentation

**Fig. 1:**
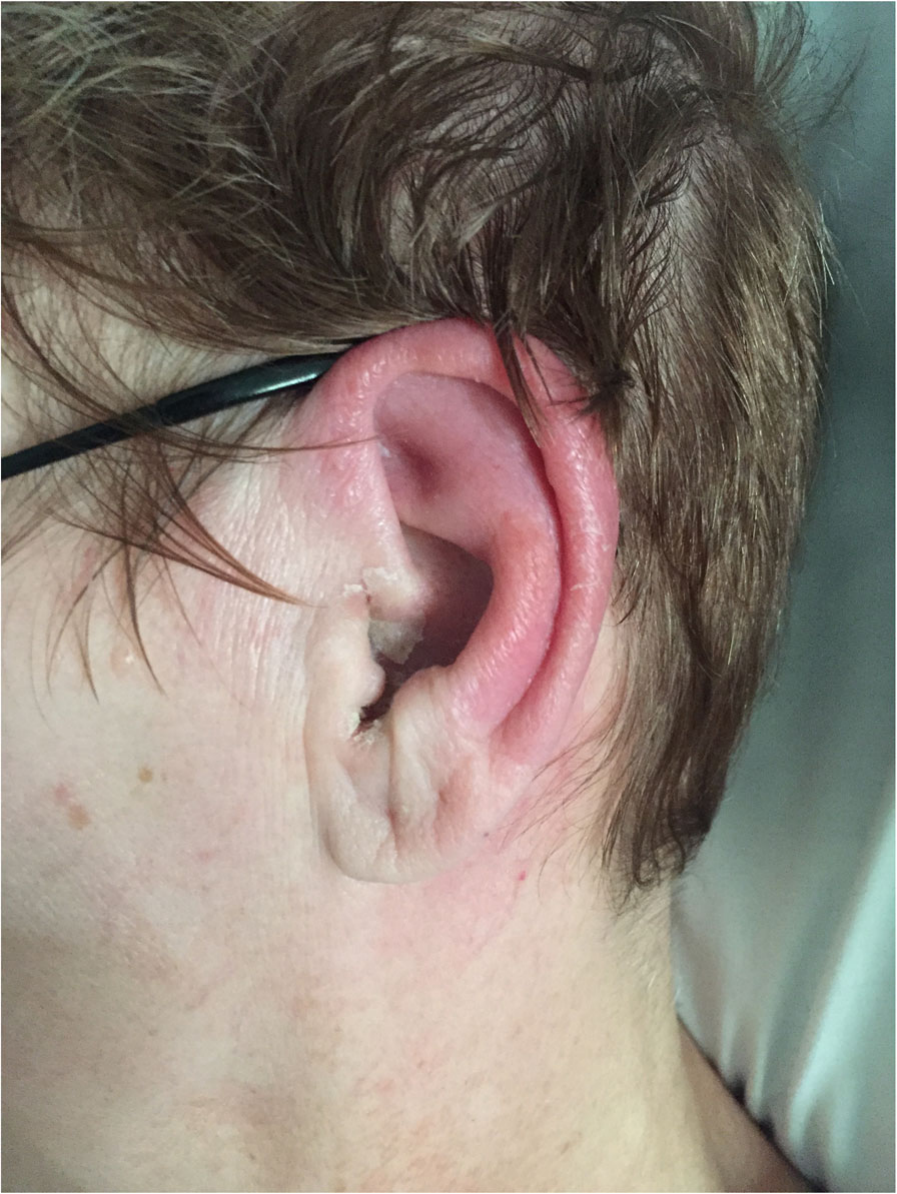
Classic picture of otitis externa with perichondritis, evidenced by erythema of the auricle with sparing of the lobule

A 57-year-old Caucasian female presented with a 4-day history of worsening left ear pain. Her symptoms began with left otalgia and otorrhea which progressed to helical erythema, prompting a visit to the emergency department (ED). The ED physician noted erythema of her left auricle and swelling of the left auditory meatus. The tympanic membrane was not visualized due to stenosis of the external auditory canal (EAC). The patient was initiated on intravenous (IV) levofloxacin and otic ofloxacin. Our otolaryngology service observed erythema of the auricle with sparing of the lobule (Fig. 1). We amended the diagnosis to be otitis externa with perichondritis and recommended otic ciprofloxacin-hydrocortisone, IV vancomycin, and ciprofloxacin. The patient had marked improvement and was discharged on an oral and otic fluoroquinolone. This clinical image was approved by the IRB at the Philadelphia College of Osteopathic Medicine.

## Diagnosis

### Otitis externa with perichondritis

Perichondritis is inflammation of the ear cartilaginous framework, often sparing the fatty lobule. When the cartilage becomes involved, including abscess formation and cartilage cavitation, the term chondritis is used [1]. Chondritis and perichondritis may result from trauma or direct extension from otitis externa. Common infectious etiologies include *P. aeruginosa*, *S. aureus*, *E. coli*, and *Proteus* species, with *P. aeruginosa* being the most common [2, 3]. If bilateral or recurrent episodes occur, workup for relapsing polychondritis should be entertained [1, 4].

Perichondritis is a clinical diagnosis made via physical examination [1, 2]. The classic finding of perichondritis is erythema and edema with sparing of the fatty lobule, which lacks any cartilaginous structure [1]. This key exam finding helps to distinguish perichondritis from otitis externa. Management of perichondritis depends on the etiology. Antibiotic therapy is the mainstay treatment with anti-pseudomonal coverage with consideration of surgical incision and drainage if abscess or hematoma is present to prevent long-term deformity [5].

## Data Availability

Not applicable

## References

[1] Pattanaik S (2009). Effective, simple treatment for perichondritis and pinna haematoma. J Laryngol Otol.

[2] Prasad HK, Sreedharan S, Prasad HS, Meyyappan MH, Harsha KS (2007). Perichondritis of the auricle and its management. J Laryngol Otol.

[3] Yahalom S, Eliashar R (2003). Perichondritis: a complication of piercing auricular cartilage. Postgrad Med J.

[4] Cristina OL, Sarv M, Claudia M (2017). Relapsing polychondritis - presenting as a recurrent auricular perichondritis-case report. Glob J Oto.

[5] Noel SB, Scallan P, Meadors MC, Meek TJ, Pankey GA (1989). Treatment of *Pseudomonas aeruginosa* auricular perichondritis with oral ciprofloxacin. J Dermatol Surg Oncol.

